# Expression of infectious murine leukemia viruses by RAW264.7 cells, a potential complication for studies with a widely used mouse macrophage cell line

**DOI:** 10.1186/1742-4690-5-1

**Published:** 2008-01-04

**Authors:** Janet W Hartley, Leonard H Evans, Kim Y Green, Zohreh Naghashfar, Alfonso R Macias, Patricia M Zerfas, Jerrold M Ward

**Affiliations:** 1 Laboratory of Immunopathology, NIAID, NIH, Bethesda, MD 20892, USA; 2 Laboratory of Persistent Viral Diseases, Rocky Mountain Laboratories, NIAID, NIH, Hamilton, MT 59840, USA; 3 Laboratory of Infectious Diseases, NIAID, NIH, Bethesda, MD 20892, USA; 4 Division of Veterinary Resources, Office of Research Services, Office of the Director, NIH, Bethesda, MD 20892, USA; 5 Comparative Medicine Branch, NIAID, NIH, Bethesda, MD 20892-8135, USA

## Abstract

The mouse macrophage-like cell line RAW264.7, the most commonly used mouse macrophage cell line in medical research, was originally reported to be free of replication-competent murine leukemia virus (MuLV) despite its origin in a tumor induced by Abelson MuLV containing Moloney MuLV as helper virus. As currently available, however, we find that it produces significant levels of ecotropic MuLV with the biologic features of the Moloney isolate and also MuLV of the polytropic or MCF class. Newborn mice developed lymphoma following inoculation with the MuLV mixture expressed by these cells. These findings should be considered in interpretation of increasingly widespread use of these cells for propagation of other viruses, studies of biological responses to virus infection and use in RNA interference and cell signalling studies.

## Background

In contrast to most other mouse-derived cell cultures, the macrophage-like cell line RAW264.7 [1] supports replication of murine noroviruses and is widely used for this purpose [2]. Further, in studies of a mouse model of severe respiratory disease, RAW264.7 was found to be uniquely efficient for propagation of the causative agent, pneumonia virus of mice, and for measuring infection-related proinflammatory mediators [3]. In addition, because of ease of cell propagation, high efficiency for DNA transfection, sensitivity to RNA interference [4], possession of receptors for many relevant ligands, and other properties, RAW264.7 has been chosen by the Alliance for Cellular Signaling as the primary experimental system for their large-scale study of signaling pathways [5]. The RAW264.7 cell line was derived about 30 years ago from a tumor developing in a BAB/14 mouse, a BALB/c IgH congenic strain, inoculated with Abelson murine leukemia virus (MuLV), a defective transforming virus containing the v-abl tyrosine kinase oncogene, and replication-competent Moloney (Mo-MuLV) that served as helper virus [1]. At the time it was described, tests for presence of replication competent virus were negative and cells in the American Type Culture Collection repository (ATCC TIB-71) were so designated until recently. As far as we can determine, ATCC is the major if not sole, commercial source of this cell line. To date, a Pubmed retrieval lists over 1500 publications that have used the RAW264.7 cell line in the research reported.

## Results and conclusion

**Figure 1:**
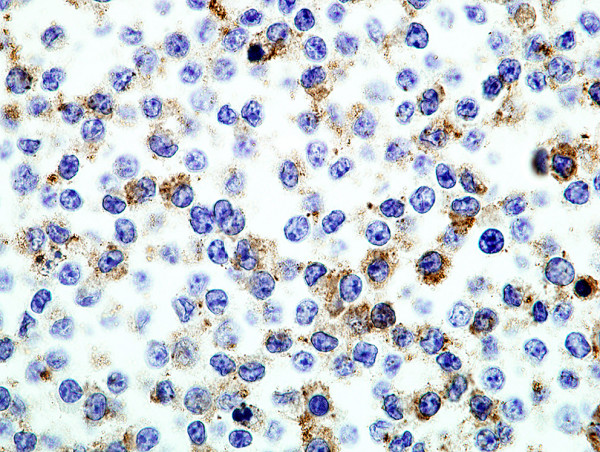
MuLV p30 expressed by RAW264.7 cells in a formalin-fixed, paraffin-embedded cell pellet. (Avidin biotin IHC, ×1000).

In the course of evaluating RAW264.7 cells for use in isolation and propagation of new isolates of murine norovirus, electron microscopy revealed particles with C-type morphology (data not shown). To look for expression of MuLV-encoded protein, RAW264.7 cell pellets were fixed in formalin and embedded in paraffin; after antigen retrieval using proteinase K, sections were stained by avidin-biotin immunohistochemistry (IHC) using group-reactive goat anti-Rauscher MuLV p30 antibody (from the Division of Cancer Cause and Prevention, NCI, Frederick, MD and obtained from Dr. Sandra Ruscetti). Abundant expression of MuLV p30 was revealed (Figure 1). Cell surface expression of MuLV gp70 protein (SU) was revealed by immunofluorescence assay using the broadly reactive monoclonal antibody 83A25 [6] (data not shown).

In tests for the presence of infectious MuLV, cell-free harvests of RAW264.7 supernatants from two separate acquisitions from ATCC were positive using the XC plaque assay [7] in SC-1 cells [8], indicating the presence of virus of the ecotropic MuLV class. Titers of several independent harvests ranged from 10^1.2 ^to 10^4.2 ^XC plaque-forming units (pfu) per ml. Lower titers were associated with high density cell growth and consequent low pH (data not shown).

Infection of cell cultures of various mouse strains by most ecotropic MuLVs is regulated by the *Fv1 *genotype of the mouse and the tropism of the virus. Thus, *Fv1*^*n *^cells are permissive for N-tropic and partially restrictive for B-tropic MuLVs; the reciprocal pattern is observed for cells of *Fv1*^*b *^mice. A few isolates with long laboratory passage histories, including Mo-MuLV, are not restricted by either allele and are termed NB-tropic. XC plaque titration of RAW264.7 supernatant in NIH3T3 (*Fv1*^*n*^) and BALB3T3 (*Fv1*^*b*^) cells gave essentially identical titers (10^4.1 ^and 10^4.2^, respectively) indicating NB-tropism. Additionally, focal immunofluoresence assays (IFA) [9] utilizing monoclonal hybridoma antibody mAb538, specifically reactive with the Mo-MuLV envelope (SU) protein [10], were positive in NIH3T3 cells and to about 1000-fold lower titer in *Mus dunni *cells, confirming the presence of a virus with ecotropic Mo-MuLV biological properties. Why earlier tests failed to detect ecotropic MuLV in RAW264.7 cells is unknown but possible explanations include differences in culture conditions and the health of the cells when assayed or in sensitivity of the tests used in different laboratories at different times.

It is well established that mouse-passaged ecotropic MuLV stocks, like the Mo-MuLV helper virus used in establishing RAW264.7 [1], frequently contain MuLV of other classes – xenotropic and recombinant MuLVs that result from interaction between ecotropic sequences and polytropic, also referred to as MCF, proviral sequences. Indeed, isolates of these classes were reported in a stock of Abelson MuLV complex [11]. Comparative IFA titrations of a RAW264.7 supernatant were performed in NIH3T3 cells using anti-Mo-MuLV mAb538 and two mAbs that specifically detect polytropic MuLV SU antigen: 514, reactive with all tested polytropic MuLVs [11], and HY7, reactive with certain polytropic subsets [12,13]. Titer estimates of 10^4 ^and 10^4.5 ^ffu per ml were obtained for polytropic and Moloney ecotropic MuLVs, respectively. Negative results with mAbs reactive with xenotropic MuLVs indicated no significant population of this class in RAW264.7 supernatants (data not shown). Thus, RAW264.7 cells express approximately equal levels of ecotropic Moloney-like and polytropic MCF MuLVs, with some variation in titer probably depending on culture conditions.

Inoculation of neonatal mice of sensitive strains with Mo-MuLV results in development of almost exclusively T cell lymphoblastic lymphomas (LL), mostly of thymic origin. To determine the pathogenic potential of harvests from RAW264.7 cells, we inoculated 1–2 day old Cr:NIH(S) (NIH Swiss) and BALB/cAnNCr (BALB/c) mice, 0.02 ml intraperitoneally and in the region of the thymus with a filtered supernatant of RAW264.7 cells or 264.7 SC-1, a harvest of SC-1 cells infected with RAW264.7 supernatant and passaged twice. For comparison, mice were similarly inoculated with Mo-MuLV (molecularly cloned and propagated in SC-1 cells). Controls were uninoculated mice of the same strains. Mice were obtained from the Division of Cancer Treatment, NCI, Frederick, MD and studied under NIAID Animal Care and Use Committee approved protocols and housing. Mice were observed for 8 to 12 months and necropsied when signs of splenomegaly, lymphadenopathy, labored breathing or lethargy were noted or the experiment was terminated at 12 months. Diagnosis was based on gross findings, microscopic examination of H&E stained formalin fixed, paraffin embedded tissues or studied by IHC using the anti-p30 antibody, anti-CD3 for T-cell lineage identification (DAKO Corporation, Carpinteria, CA Catalog # A452), and anti-PAX5 for B-cell lineage (Goat anti-Pax 5, Santa Cruz Biotechnology, Santa Cruz, CA, Catalog #sc-1974) [14]. Criteria for histopathological diagnosis were as described [15].

**Figure 2:**
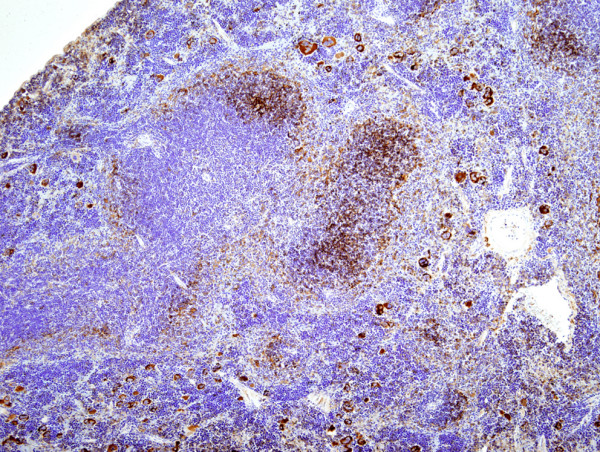
MuLV p30 expression in spleen 8 weeks post injection of 264.7-MuLV, SC-1 prior to lymphoma development (IHC, hematoxylin, ×100).

**Figure 3:**
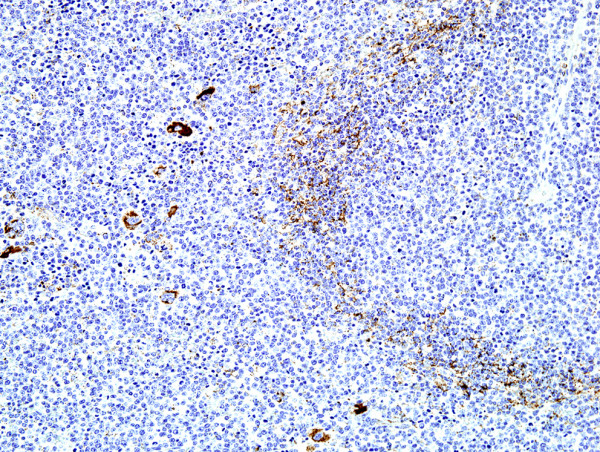
MuLV p30 in splenic follicular B-cells and megakaryocytes in a mouse injected with 264.7-MuLV and that developed thymic lymphoma at 119 days (IHC, hematoxylin, ×200).

**Figure 4:**
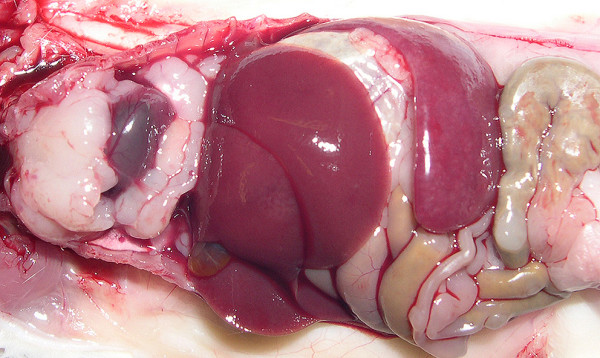
Thymic lymphoma induced by 264.7-MuLV, 164 days post injection. Note large spleen.

**Figure 5:**
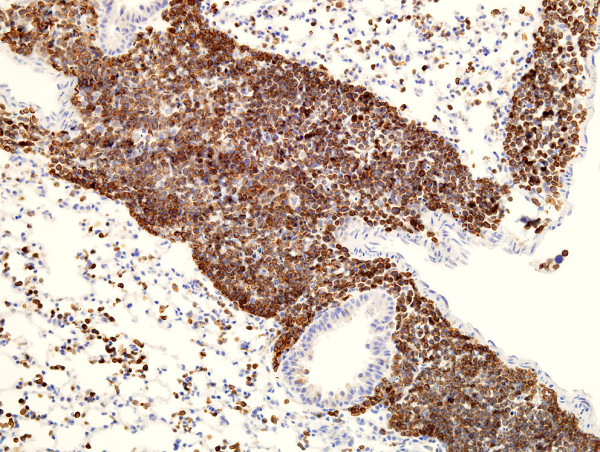
Thymic lymphoma in 264.7-MuLV infected mouse showing CD3^+ ^lymphoma cells in lung metastases (IHC, hematoxylin, ×200).

**Figure 6:**
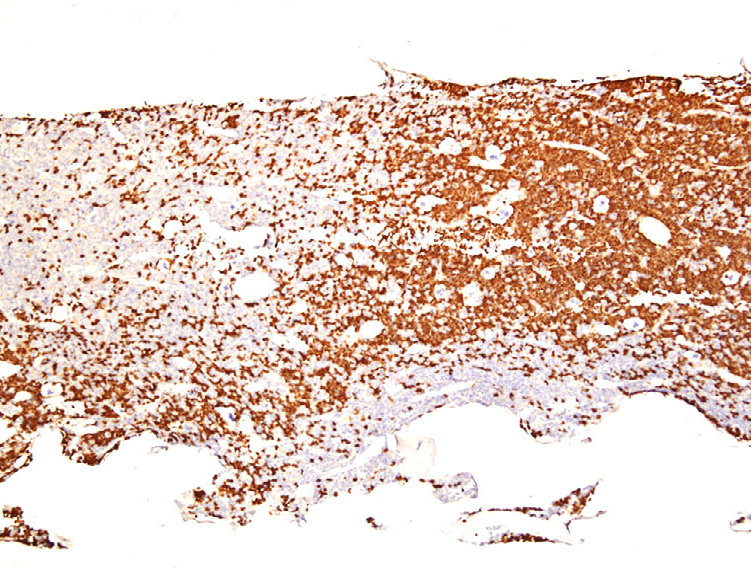
PAX5 expression in bone marrow metastases of a B-cell lymphoma induced by RAW 264.7-MuLV (IHC, hematoxylin, ×100).

As shown in Table 1, there were no significant differences in tumor incidence between NIH Swiss and BALB/c recipients but virus dose was clearly an important variable for those inoculated with the viruses produced by RAW264.7 cells (264.7MuLVmix), with higher concentrations tending to give shorter latencies and more diversity in pathology. Mo-MuLV induced mostly T-cell LL, characterized histologically by diffuse growth of CD3^+ ^T-cell lymphoblasts usually originating in the thymus and metastasizing to the spleen, liver and other organs. Most mice presented with significantly enlarged thymus (16/19), spleen weights of over 600 mg (18/19) and variable lymphadenopathy. Mice injected with 264.7-MuLVmix expressed MuLV p30 in spleen (Figure 2), megakaryocytes, and many tissues prior to tumor development and in all lymphomas examined (e.g., Figure 3). Splenomegaly and lymphadenopathy were variable with spleen weights ranging from 100 to 1800 mg. The majority of hematopoietic neoplasms were classified as LL (26/34). Of these, 19 were of T-cell origin, CD3^+ ^and PAX5^-^; 13 were associated with enlarged thymus (Figure 4). In contrast to CD3+ Mo-MuLV-induced LL (Figure 5), the remaining seven LL were of B-cell origin, based on PAX5^+ ^IHC (Figure 6) and CD3 negativity. Spleen weights ranged from 450 to 770 mg and lymph node size was variable. In addition, one BALB/c mouse had an early splenic marginal zone B cell lymphoma (MZL) as well as early thymic T-LL. To our knowledge, spontaneous splenic MZL has not been reported in this strain. There are sporadic reports of exogenous virus induction of B cell lymphomas in mice by a variety of ecotropic and polytropic MuLVs [16,17], none to our knowledge involving Mo-MuLV except for pre-B LL induction in *Eμ-myc *transgenic mice [18]. Pre-B, immature B cells and plasma cells are the target cells of Abelson MuLV [19] and mice infected with the LP-BM5 complex, which includes a defective immunodeficiency-inducing virus, develop transplantable clonal B-cell populations [20]. We did not detect Abelson transforming capacity in tissue culture assays (data not shown), but it is conceivable that B-cell LLs found in our study might be causally related to the Abelson genome present in RAW264.7 cells. Alternatively, B cell pathology may be associated with the polytropic virus population present in RAW264.7 supernatants, likely in synergy with Mo-MuLV. Further testing with cloned virus preparations would be necessary to resolve these possibilities.

**Table 1:** Comparative Pathogenicity of 264.7MuLVs and Mo-MuLV in Mice

					**Diagnosis**^3^				
**Mouse Strain**	**Inoculum**	**Virus dose**^1^	**#pos/#inoc**^2^	**Latency **(days)	**% positive**	**LL-T**	**LL-B**	**Erythroid**	**Other**
NIH Swiss	Mo-MuLV	10^4.4^	6/6	87 +/- 16	100	4	0	2^4^	0
		10^2.2^	10/10	93 +/- 13	100	10	0	0	0
	RAW264.7 cell-free supernatant	10^1.0^	5/7	259 +/- 68	71	3	0	1	1^5^
		10^2.5^	12/15	163 +/- 68	80	5	4	3	0
	264.7-MuLV, SC-1 passage	10^2.2^	3/3	146 +/- 24	100	2	1	0	0
	None	-	0/5						
BALB/c	Mo-MuLV	10^4.4^	5/5	98 +/- 14	100	5	0	0	0
	RAW264.7 cell-free supernatant	10^1.0^	2/2	255	100	1	0	1	0
		10^2.5^	7/12	168 +/- 53	58	4	2^4^	1	0
	264.7-MuLV SC-1 passage	10^2.2^	5/5	216 +/- 84	100	4	0	0	1^6^
	None	-	0/4						

^1 ^pfu/mouse of ecotropic MuLV, based on XC plaque titration in SC-1 cells; polytropic MuLV titer not determined.

^2 ^number of mice positive for hematopoietic disease/number inoculated.

^3 ^LL, lymphoblastic lymphoma;, T, T-cell lineage; B, B-cell lineage; erythroid, erythroleukemia (PAX5 and CD3 negative).

^4 ^One case also had early Thymic T-LL.

^5 ^Mast cell tumor, spleen and bone marrow (366d post-inoculation).

^6 ^Early splenic marginal zone lymphoma (295d post-inoculation) ; also very early T-LL in thymus.

Six of 34 264.7MuLVmix- and 2 of 16 MoMuLV-induced tumors were non-lymphoid and diagnosed as erythroleukemia based on splenomegaly with a high frequency of erythroid cells and lack of reactivity with CD3 and PAX5. These neoplasms, not usually seen following Mo-MuLV infection, may be related to the generalized hyperplasia of hematopoietic lineages, including erythroid, reported in pre-leukemic Mo-MuLV-infected mice [21].

A further unusual finding was a mast cell tumor, a rarely seen mouse neoplasm that cannot unequivocally be considered related to the virus inoculation.

As shown in this report, RAW264.7 cells as currently available from ATCC express ecotropic and polytropic MuLVs. The ecotropic virus has biological properties of the Mo-MuLV helper virus of the Abelson virus complex that induced the tumor from which the cell line derived. Cell-free culture supernatants containing the mixed virus population induced hematopoietic disease in newborn mice, primarily LL that were mostly of T cell type, as is characteristic of Mo-MuLV lymphomagenesis, but also some of B cell origin. This tumor-inducing potential may confound pathogenicity testing of unrelated viruses propagated in RAW264.7, especially in newborn mice. Adult mice are susceptible to infection by Mo- and other MuLVs, however [22,23], and mixed infection of MuLVs with related and unrelated viruses may have effects not directly attributable to lymphomagenicity. For example, radiation-induced RadLV enhances expression of MHC Class I genes [24] as does Mo-MuLV in cell culture [25]. Synergism between different retroviruses of low pathogenicity induces a rapidly fatal neurological disease [26], ecotropic MuLV potentiates LDV-related paralytic disease [27] and Mo-MuLV potentiates polyomavirus-induced runting syndrome [28].

Ecotropic MuLV infection of adult mice has been shown to increase B-cell proliferation, serum immunoglobulin M levels and expression of transcripts associated with B cell activation [23]. Further, infection of bone marrow cells with MoMuLV or bone marrow and primary B cells by Abelson MuLV induces expression of activation-induced cytidine deaminase (AID) by activating NFκB [29,30]. Ectopic expression of AID can result in generalized somatic hypermutation [31].

Such illustrations of unanticipated consequences of MuLV infection as well as conceivable disruptive effects of MuLV replication, including integration into cellular DNA and cell surface expression of MuLV antigens, suggest caution in experimental design and data interpretation in studies utilizing RAW264.7 cells.

## Abbreviations

MuLV: Murine leukemia virus; 

Mo-MuLV: Moloney MuLV; 

IFA: Immunofluorescence focus assay; 

LL: Lymphoblastic lymphoma; 

IHC: Immunohistochemistry.

## Competing interests

The author(s) declare that they have no competing interests.

## Authors' contributions

JMW, KYG and JWH conceived and designed the study. JMW performed histopathological observations and carried out IHC studies. LHE carried out focal IFA. ZN performed cell culture and viral quantitation studies. PZ performed electron microscopy and first found the virus particles. AM, JWH and JMW carried out mouse studies. JWH and JMW drafted the paper.
